# Motivational and myopic mechanisms underlying dopamine medication-induced impulsive-compulsive behaviors in Parkinson's disease

**DOI:** 10.3389/fnbeh.2022.949406

**Published:** 2023-01-18

**Authors:** Andrew Dawson, Paola Ortelli, Adrian Carter, Davide Ferrazzoli, Nadeeka N. Dissanayaka, Andrew Evans, Yann Chye, Valentina Lorenzetti, Giuseppe Frazzitta, Murat Yücel

**Affiliations:** ^1^Monash Institute of Cognitive and Clinical Neurosciences, Monash University, Clayton, VIC, Australia; ^2^Department of Neurorehabilitation, Hospital of Vipiteno (SABES-ASDAA), Lehrkrankenhaus der Paracelsus Medizinischen Privatuniversität, Vipiteno-Sterzing, Italy; ^3^Department of Movement Disorders and Brain Injury Rehabilitation, “Moriggia-Pelascini” Hospital, Como, Italy; ^4^Faculty of Medicine, University of Queensland Centre for Clinical Research, The University of Queensland, Herston, QLD, Australia; ^5^Department of Neurology, Royal Brisbane and Women's Hospital, Herston, QLD, Australia; ^6^School of Psychology, The University of Queensland, St. Lucia, QLD, Australia; ^7^Department of Movement Disorders, The Royal Melbourne Hospital, Parkville, VIC, Australia; ^8^School of Psychology, Australian Catholic University, Fitzroy, VIC, Australia; ^9^MIRT ParkProject, Livorno, Italy

**Keywords:** Parkinson's disease, dopamine replacement therapy, addiction, motivation, impulsivity, aggression

## Abstract

**Introduction:**

Dopaminergic medications can trigger impulsive-compulsive behaviors (ICBs) in pre-disposed patients with Parkinson's disease (PD), but what this implies on a neurocognitive level is unclear. Previous findings highlighted potentially exacerbated incentive motivation (willingness to work for rewards) and choice impulsivity (preferring smaller, immediate rewards over larger, delayed rewards) in PD patients with ICBs (PD + ICBs).

**Methods:**

To deeply understand this evidence, we studied 24 PD + ICBs and 28 PD patients without ICBs (PD-ICBs). First of all, patients underwent the assessment of impulsivity traits, mood, anxiety, and addiction condition. We further administered robust objective and subjective measures of specific aspects of motivation. Finally, we explored whether these processes might link to any heightened antisocial behavior (aggression and risky driving) in PD + ICBs.

**Results:**

High levels of positive urgency trait characterized PD + ICBs. They choose to exert more effort for rewards under the conditions of low and medium reward probability and as reward magnitude increases. Findings on choice impulsivity show a great tendency to delay discounting in PD + ICBs, other than a high correlation between delay and probability discounting. In addition, we found what appears to be the first evidence of heightened reactive aggression in PD patients with ICBs. Exacerbated incentive motivation and delay discounting trended toward positively predicting reactive aggression in PD + ICBs.

**Discussion:**

Our promising results suggest that there might be immense value in future large-scale studies adopting a transdiagnostic neurocognitive endophenotype approach to understanding and predicting the addictive and aggressive behaviors that can arise from dopaminergic medication in PD.

## 1. Introduction

Dopamine replacement therapy (DRT) represents the cornerstone pharmacological treatment for Parkinson's disease (PD). While in early PD, dopaminergic therapy is usually effective in improving the dominant motor features of the disease, its effectiveness in the medium-advanced stages tends to decrease. Furthermore, the potential onset of DRT-related side effects, such as motor fluctuations, dyskinesia, painful dystonia, dopamine dysregulation syndrome, and impulsive–compulsive behaviors (ICBs), conspires, over time, to reduce the overall tolerability of pharmacological therapies (Chapuis et al., 2005). The latter could be defined as motivational side effects. They include motor stereotypies, such as punding (repetitive, stereotypical, and mindless behavior, e.g., collecting, arranging, or dismantling), appetitive behaviors, such as hypersexuality, pathological gambling, compulsive shopping, and binge eating, as well as compulsive use of excessive DRT, termed “Dopamine Dysregulation Syndrome” (Lawrence et al., 2003; Voon et al., 2007). The impact of these insidious disturbances in PD is relevant as they affect around 14% of patients (Weintraub et al., 2010). Other than in patients with PD, ICBs exist in both the general population (Kessler et al., 2005) and adult psychiatric cohorts (Grant et al., 2005). In this context, the prevalence of ICBs varies worldwide and is likely influenced by culture. For example, problematic gambling rates in the general population vary between 0.2% in Norway and 5.3% in Hong Kong (Hodgins et al., 2011) and 1.9% in the United States (Welte et al., 2001), and the point prevalence of compulsive buying has been estimated to be 5.8% in the United States (Koran et al., 2006). In the population with PD, the association between DRT, particularly dopamine agonists (DAs) in higher dosages, and ICBs have raised much concerns (Giladi et al., 2007; Zhang et al., 2014). Rodent studies support a role for the D2-receptor class in the motivational effects of DRT since both D2-like and D2/D3 receptor agonists have consistently shown reinforcing properties in intact animals (Cenci et al., 2015). Translating these results, it has been postulated that ICBs in PD reflect overvaluation of rewards, resulting from excessive dopaminergic transmission in the ventral striatum (Gatto and Aldinio, 2019). Other results (Housden et al., 2010) contradict this vision, being more consistent with a model in which excessive dopaminergic transmission induces a strong preference for immediate over future rewards, thus driving maladaptive behaviors in PD patients with ICBs. As a matter of fact, how dopaminergic modulation triggers these aberrant behaviors or what predisposes some individuals to develop these alterations remains unknown. A previous systematic review of the literature (Dawson et al., 2018) concerning the neuropsychological features of PD patients with ICBs (PD + ICBs) has concluded that only two domains exhibit any consistency in terms of exacerbation or dysfunction in PD + ICBs: *incentive motivation* (willingness to work for rewards, especially when reward receipt is unlikely) and *choice impulsivity* (a preference for smaller, immediate rewards over larger, delayed rewards). Evidence of dysfunction in reinforcement learning, information sampling, and risky decision-making under uncertainty was weak, and we found negligible evidence of impairment in motor or cognitive control in PD + ICBs.

The potential implication of incentive motivation is consistent with claims that mesolimbic dopamine in the brain's reward pathway (a) creates an imbalance between the evaluation of effort and reward, increasing the willingness to work toward rewards (Salamone and Correa, 2012) and (b) promotes a “wanting” condition more tightly linked to reward than connected to a “cognitive” goal (Berridge and Robinson, 2016). The latter view suggests that the intrinsic pulsatile nature of DRT could promote addiction and, more generally, ICBs in predisposed patients with PD (Berridge and Robinson, 2016). Similarly, *choice impulsivity* appears dopaminergically modulated (Pine et al., 2010) and is an extremely robust behavioral marker of addiction and ICBs (Bickel et al., 2014). *Choice impulsivity* refers to maladaptive decision-making in which smaller-sooner rewards could be favored in comparison with larger-later ones, following a function in which the temporal variable became crucial: the so-called temporal discounting (Rung et al., 2019). However, there are only few studies aimed at deepening both of these domains and only a few bodies of evidence exist about exacerbated incentive motivation (Terenzi et al., 2018) and choice impulsivity (Martini et al., 2018) in PD + ICBs patients (Dawson et al., 2018). We seek to clarify the role of incentive motivation and choice impulsivity by comparing the performance of PD + ICBs and PD patients without ICBs (PD-ICBs) on objective and subjective measures of these processes. We expect significant PD + ICBs exacerbation on both sets of measures, relative to PD-ICBs.

We, therefore, explore whether any exacerbated incentive motivation and choice impulsivity in PD + ICBs could be associated with higher levels of antisocial behavior in those patients. In particular, we refer here to aggressive and risky driving behaviors, as they have been previously described in patients suffering from ICB. In fact, previous studies have shown that reactive, but not proactive, aggression characterizes PD + ICBs patients (Djamshidian et al., 2011). Risky driving has also been observed in some patients with PD consuming large amounts of dopaminergic medication (Avanzi et al., 2008), as well as in those with major motor vehicle accidents (Ando et al., 2018). It is not yet clear to what extent these antisocial behaviors could be considered typical in PD + ICBs or whether they represent simply a rare clinical manifestation. For this purpose, we compare a subset of PD + ICBs (*n* = 16) and PD-ICBs (*n* = 21) on objective and subjective measures of reactive aggression and risky driving, before attempting to account for any exacerbated antisociality in PD + ICBs in terms of enhanced incentive motivation or choice impulsivity.

## 2. Experimental procedures

### 2.1. Patients and methods

Participants were recruited from a major provincial hospital in Italy and major metropolitan hospitals and community practices in Australia.

Inclusion criteria were (1) diagnosis of idiopathic PD, classified among the 1–3 stages of Hoehn & Yahr's (H&Y) scale and (2) pharmacological treatment with dopaminergic drugs. Exclusion criteria were (1) presence of comorbid neurological and/or psychiatric conditions; (2) history of addiction and/or obsessive-compulsive disorders, arising before PD diagnosis; (3) deep brain stimulation; and (4) cognitive impairment and/or dementia.

Based on the presence or absence of ICBs [investigated through Parkinson's Impulse-Control Scale (PICS) score, as mentioned earlier], patients were recruited for generating two groups: 24 participants composed the “PD with ICBs” group (PD + ICBS) and 27 “matched” patients composed the “PD without ICBs” group (PD-ICBS). Of these 52 patients, 37 were Italian, and 15 were Australian.

Participants provided informed consent, and they received remuneration for their participation, whose total amount depend on individual task performance. Consistently with local ethical practice, patients from Italy were reimbursed with food and beverage tokens only. Ethics approval was obtained from the Monash University Human Research Ethics Committee (CF16/129-2016000054) and the provincial ethics committee in Italy (*Comitato Etico interaziendale delle Province di Lecco–Como–Sondrio*).

### 2.2. Measures

Bilingual authors (PO and DF) generated Italian versions of all tasks and then back-translated these versions for verification. Some questionnaires (denoted by an asterisk) were translated from English to Italian (by VL) and back-translated and verified by PO and DF. Refer to Supplementary material for more details on all measures.

#### 2.2.1. Clinical assessment

Age, sex, age at disease onset, H&Y stage, and Unified Parkinson's Disease Rating Scale (UPDRS) III had been collected. Levodopa equivalent daily dose (LEDD) was calculated using standard criteria (Tomlinson et al., 2010), and the assumption of DAs was registered. The presence and severity of any ICBs were measured with the *PICS*, a semi-structured interview-based tool aimed to detect ICBs (Okai et al., 2016).

From a psychiatric point of view, Australian patients underwent the *Mini-International Neuropsychiatric Interview Version 5*, while Italian patients underwent the *Structured Clinical Interview for DSM Disorders-IV*.

Since, in PD + ICBs, there is a tendency toward higher levels of anxious and depressive symptoms relative to PD-ICBs (Voon et al., 2011), all patients filled in some self-administered scales: the *Geriatric Depression Scale-15* (*GDS*) and the *Parkinson Anxiety Scale* (*PAS*).

As ICBs in PD are associated with current cigarette smoking (Weintraub et al., 2010), the *Fagerström Test for Nicotine Dependence* (*FAGER*) has been administered, while, to screen patients for other potential abuse of substances, the *Alcohol Use Disorder Identification Test* (*AUDIT*) and the *Drug Abuse Screening Test-10* (*DAST*) were adopted.

We deeply investigate the Urgency-Premeditation-Perseverance-Sensation Seeking-Positive Urgency (UPPS-P) impulsive behavior scale, which revealed five specific facets of impulsivity, namely, sensation seeking, lack of premeditation, lack of perseverance, negative urgency, and positive urgency (Lynam et al., 2006).

Finally, from a neuropsychological point of view, patients underwent Montreal Cognitive Assessment (MoCA) for assessing global cognition.

#### 2.2.2. Experimental procedure: Neurocognitive tests and questionnaires

As depicted in the Supplementary material, we studied four different neuropsychological domains of ICBs through a computerized task and a self-administered computerized questionnaire for each area of interest. The Supplementary material section offers an overview of each task and questionnaire.

#### 2.2.3. Incentive motivation

1. *Effort Expenditure for Rewards Task* (*EEfRT*) (Treadway et al., 2009). Participants choose between an “easy” and “hard” task, both of which potentially yield rewards, on each trial. The following variables are most important in predicting the choice of hard task: the magnitude of the reward available for completing the hard task; the probability of reward receipt (12, 50, or 88%); group status (i.e., clinical or control); and trial number (to account for fatigue). The task was timed and it ended after 20 min, regardless of the number of completed trials.
2. *BIS/BAS* (Carver and White, 1994) is a measure of behavioral inhibition (*BIS*) and behavioral approach (*BAS*). *BAS* comprises drive, fun-seeking, and reward responsiveness subscales. The drive subscale was used as a subjective measure of incentive motivation.

#### 2.2.4. Choice impulsivity

3. *Delay and Probability Discounting Task* (*DPDT*) (Richards et al., 1999). The *DPDT* presents and modifies over time choices between smaller and larger rewards. Smaller rewards can be immediate or certain. Larger rewards can be delayed or probabilistic. Indifference points are determined for each participant. Averaging these yields separate delay and probability discounting parameters for each participant.
4. *Monetary Choice Questionnaire* (*MCQ*) (Kirby et al., 1999). The *MCQ* poses choices between hypothetical smaller-immediate sums and larger-later sums. Participants' choices are used to calculate their discounting rate *k*.

#### 2.2.5. Reactive aggression

5. *Point Subtraction Aggression Paradigm* (*PSAP*) (Cherek et al., 1997). In *PSAP*, participants play against an anonymous (actually fictitious) online opponent to earn money. By pressing different buttons, participants can choose to earn points, deduct points from their “opponent” in response to transparent “opponent” point “theft,” or protect points temporarily. Participants' proportion of retaliatory responses is the key outcome measure. To reduce participant burden, patients completed the *PSAP* in 12 min instead of the usual 25 min (Golomb et al., 2007).
6. *Buss-Perry Aggression Questionnaire* (*BPAQ*) (Buss and Perry, 1992). *BPAQ* measures *four* dimensions of aggression, namely, physical aggression, verbal aggression, anger, and hostility.

#### 2.2.6. Risky driving

7. *Stoplight Task* (Chein et al., 2011). The *stoplight* is a simple driving task requiring participants to pass through 32 intersections to reach their target destination in <8 min. As intersections with yellow traffic lights approach, participants can brake and temporarily stop using the space bar or proceed through the intersection, but risk crashing and losing a significant amount of time.
8. *Driver Behavior Questionnaire* (*DBQ*) (Lajunen et al., 2004). *DBQ* measures general driving behavior in terms of lapses, errors, ordinary violations, and aggressive violations.

### 2.3. Procedure

Eligible participants were provided with an explanatory statement before providing informed consent. Participants were confirmed to be “on” medication *via* self-report. Demographic data were then collected and neurocognitive tasks were administered in random order on a computer in a quiet room with ample breaks. Detailed instructions and a practice session preceded each task. The experimental session lasted between 3.5 and 5 h. Participants were fully debriefed upon completion.

### 2.4. Data analysis

Data were analyzed using SPSS version 23.

Central tendency and dispersion of continuous variables are reported as mean and standard deviation (SD) for demographical and neuropsychological data and as mean and standard error (SE) for neurophysiological outcomes. Descriptive statistics for categorical variables are reported as numbers and percentages. Between-group comparisons were carried out by the Mann–Whitney *U*-test for continuous variables and by the chi-square test for dichotomous variables.

*EEfRT* data were analyzed using generalized estimating equations (GEEs), where the group was the main predictor of interest together with reward probability, reward magnitude, and number of “hard task choice” trials (we named Hard-Trial Number). Other demographic variables were considered additional predictors of interest, in particular, the positive urgency trait from the *UPPS-P*; the assumption of DAs; the FAGER scores; the GDS scores; and the PAS scores. The first GEE model tested the effect of *EEfRT* task features: reward receipt probability, reward magnitude, and hard-trial number (Geaney et al., 2015). The second model included the group as the main predictor of interest, hard-trial Number, and relevant covariates. The third model tested the interaction effect of the group under low, medium, and high reward probability while accounting for hard-trial number and relevant covariates. The fourth model resembled the third model but included reward magnitude instead of reward receipt probability.

*BAS Drive* scores were analyzed using an independent samples *t*-test. For the *DPDT*, each subject's delay and probability discount rate (*k*) was separately calculated from their respective indifference points using the formula V = A/(1 + *k*D), where V is the indifference point, A is the amount of the reward, and D is the delay to reward (Richards et al., 1999). *K*-values were then log_10_ transformed to ensure normal distribution. Average log_10_*k*-values were compared between PD + ICBs and PD-ICBs groups in two ANCOVA models (one for the delay parameter and one for the probability parameter). Four separate ANCOVAs were run to examine *k* for (i) all rewards, (ii) small rewards, (iii) medium rewards, and (iv) large rewards on *MCQ*. Finally, *PSAP, BPAQ, Stoplight*, and *DBQ* were analyzed using ANCOVA with a group (PD + ICBs or PD-ICBs). The assumption of DA drugs as fixed factors, FAGER score, GDS score, PAS score, and positive urgency trait (from the *UPPS-P*) is considered in the analysis as covariates. The linear regression was employed to determine any association between (a) any PD + ICBs elevation incentive motivation and choice impulsivity; (b) covariates; and (c) any significant PD + ICBs elevation on antisociality measures.

Finally, to study whether the heightened reactive aggression observed in PD + ICBs could be accounted for in terms of exacerbated incentive motivation and/or delay discounting. Two linear regressions were conducted. As predictors of heightened laboratory aggression, the first model employed (a) the proportion of hard task choices under low and medium reward probability on the *EEfRT*; (b) the objective, log-transformed delay discounting parameter log_10_*k*; and (c) demographic covariates. We repeated this process for heightened subjective aggression.

## 3. Results

### 3.1. Clinical assessment

Table 1 describes the demographic characteristics of the participants. Age, sex, level of education, disease duration, H&Y, UPDRS-III, LEDD, and assumption of DAs were highly matched, ensuring two groups differed only for the presence of ICBs.

**Table 1:** Demographic, neurological, and clinical characteristics of participants.

	**PD** + **ICBs (*****n*** = **24)**		**PD-ICBs (*****n*** = **28)**		***t, χ^2^-values***	***P***
	***M***	***SD***	***M***	***SD***		
**Clinical features**						
Age	59.667	8.176	62.607	9.207	1.208	0.223
Gender (M/F)	19/5		16/12		2.849	0.091
Education	12.875	4.407	13.929	4.891	0.810	0.422
Years from disease onset	8.417	4.596	9.607	6.196	0.776	0.442
*UPDRS III* (ON state)	15.875	5.472	17.462	4.226	1.153	0.255
Hoehn & Yahr (ON state)	2.146	0.667	2.385	0.668	1.264	0.212
LEDD	678.667	276.027	604.464	365.015	0.815	0.419
Dopamine agonists (no/yes)	7/17		15/13		3.153	0.076
Dopamine agonists (levodopa equivalent dose mg)	226.940	142.381	210.150	116.134	0.346	0.732
**Psychiatric features**						
*AUDIT*	1.750	2.575	1.440	1.387	0.528	0.600
*DAST*	0.042	0.204	0	0	1.021	0.312
*FAGER*	0.708	1.853	0	0	1.912	0.062
*GDS*	3.739	3.250	3.304	3.169	0.459	0.648
*PAS*	11.083	9.146	14.348	10.998	1.108	0.274
*UPPS-P* negative urgency	25.826	7.303	22.182	6.681	1.744	0.088
*UPPS-P* lack of premeditation	18.913	4.926	17.046	4.603	1.313	0.196
*UPPS-P* lack of perseverance	19.348	4.238	17.273	3.453	1.796	0.080
*UPPS-P* sensation seeking	24.652	6.541	23.818	4.992	0.479	0.634
*UPPS-P* positive urgency	26.043	6.698	21.773	6.803	2.122	0.040*
Single/multiple ABs	16/8^∧^		0			
**Neuropsychological features**						
*MOCA*	25.750	2.524	26.286	2.720	0.732	0.468

MOCA, Montreal cognitive assessment test; UPDRS III, Unified Parkinson's disease rating scale (part III); LEDD, Levodopa equivalent daily dose; AUDIT, Alcohol use disorder identification test; DAST, Drug abuse screening test-10; FAGER, Fagerström test for nicotine dependence; GDS, Geriatric depression scale-15; PAS, Parkinson anxiety scale.

* Significant at p < 0.05.

∧ Single ICBs: Pathological gambling (n = 2), Hypersexuality (n = 1), Compulsive shopping or reckless spending (n = 2), Binge eating (n = 1), Dopamine dysregulation syndrome (n = 3), Punding (n = 1), Hobbyism (n = 5); Multiple ABs: 2 ABs (n = 4), 4 ABs (n = 4).

Self-administered questionnaires and scales provided comparable data among patient populations. The analysis of UPPS-P items led us to observe that the “positive urgency” trait was higher in PD + ICBs in comparison with PD-ICBs. Consequently, this trait has been inserted as a covariate for all subsequent analyses.

Finally, the MoCA score did not differ between groups.

### 3.2. Experimental procedure: Neurocognitive tests and questionnaires

#### 3.2.1. Incentive motivation

**Figure 1:**
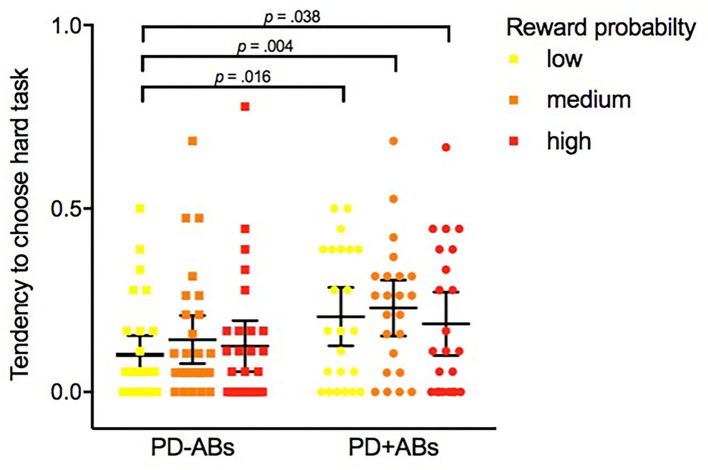
Tendency average of PD-Abs and PD + ABs to choose the hard task on the *Effort Expenditure for Rewards Task* under different levels of reward probability: low (12%), medium (50%), and high (88%).

1. *EEfRT* task. To guarantee consistency maintaining, for each patient, we consider for the analysis the first 55 trials (corresponding to the minimal number of trials completed by any participant) (Geaney et al., 2015). On average, PD + ICBs chose the hard task 11.391 times (*SD* = 9.258) across 55 trials, while PD-ICBs chose the hard task 6.786 times (*SD* = 8.112) (refer to Figure 1). Table 2 shows the results of the GEE models. Trial number and nicotine dependence score were consistently negative predictors of hard task choice across all models in which they were assessed. Higher reward magnitude also predicted hard task choice in model 1, whereas reward probability did not. ICB status did not alone predict hard task choice in model 2, but in model 3, the presence of ICB was positively predictive of hard task choice when interacting with the low, medium, and high reward probability predictors (refer also to Figure 1). Higher depressive symptoms were predictive of avoiding hard task choices in this model. Finally, ICB predicted the choice of the hard task as the reward magnitude increased (model 4).
2. BIS/BAS. PD + ICBs reported a mean BIS/*BAS* drive subscale score of 11.750 (*SD* = 2.817), while PD-ICBs reported a mean of 10.280 (*SD* = 3.247), resulting comparable *t*_(47)_ = 1.69, *p* = 0.098.

**Table 2:** Predicting hard task choice with task features, group, and demographic covariates across four models.

		***Wald χ^2^***	***B***	**SE**	***P***
Model 1	Medium probability	1.983	0.165	0.117	0.159
	High probability	0.011	0.017	0.163	0.918
	Reward magnitude	23.781	0.278	0.057	< 0.001^***^
	Trial number	17.692	−0.019	0.005	< 0.001^***^
Model 2	Group	2.965	0.851	0.494	0.085
	Trial number	15.687	−0.019	0.005	< 0.001^***^
	DAs (no/yes)	0.359	−0.235	0.392	0.549
	*FAGER*	35.537	−2.931	0.492	< 0.001^***^
	*GDS*	3.460	−0.129	0.069	0.063
	*PAS*	0.252	−0.014	0.028	0.616
	*UPPS_P_POSURG*	0.246	−0.015	0.031	0.620
Model 3	PD + AB × Low^a^	5.788	1.021	0.421	0.016^*^
	PD + AB × Medium^a^	8.256	1.140	0.397	0.004^**^
	PD-AB × Medium^a^	0.610	0.304	0.390	0.435
	PD + AB × High^a^	4.288	0.972	0.469	0.038^*^
	PD-AB × High^a^	0.334	0.252	0.435	0.563
	Trial number	16.691	−0.019	0.005	< 0.001^***^
	DAs (no/yes)	0.857	−2.35	−2.54	0.355
	*FAGER*	13.064	−2.932	0.811	< 0.001^***^
	*GDS*	8.740	−0.129	0.044	0.003^**^
	*PAS*	0.581	−0.014	0.019	0.446
	*UPPS_P_POSURG*	0.613	−0.015	0.020	0.434
Model 4	+AB × RM	20.159	0.375	0.084	< 0.001^***^
	–AB × RM	1.123	0.143	0.135	0.289
	Trial number	4.807	−0.019	0.005	< 0.001^***^
	DAs (no/yes)	0.135	−0.137	0.372	0.713
	*FAGER*	37.640	−2.986	0.487	< 0.001^***^
	*GDS*	3.137	−0.118	0.067	0.077
	*PAS*	0.467	−0.019	0.028	0.494
	*UPPS_P_POSURG*	0.093	−0.009	0.031	0.761

DAs, dopamine agonists; FAGER, Fagerström test for nicotine dependence; GDS, Geriatric depression scale-15; PAS, Parkinson anxiety scale; UPPS_P_POSURG, UPPS-P positive urgency; RM, reward magnitude.

* p < 0.05,

** p < 0.01,

*** p < 0.001.

a Low, low reward probability; Medium, medium reward probability; High, high reward probability. Interaction effect tested relative to PD-AB × low reward probability.

Fatigue prevented one PD + ABs from completing the EEfRT.

Two PD + ABs and two PD-ABs failed to complete the BAS drive subscale.

#### 3.2.2. Choice impulsivity

Table 3 displays participants' delay and probability discounting parameters derived from the *DPDT* and *MCQ*.

3. DPDT. Groups differed significantly on the delay [*F*_(1,34)_ = 4.509, *p* = 0.041, η^2^ = 0.117] but not probability discounting parameters log_10_*k* [*F*_(1,34)_ = 0.767, *p* = 0.387], with PD + ICBs exhibiting steeper delay discounting.
4. MCQ. No differences emerged [*F*_(1,29)_ = 2.942, 0.945, 3.068, and 2.377, respectively; *p* range = 0.0900–0.339].

**Table 3:** Groups' performance on choice impulsivity measures.

		**PD** + **ICBs (*****n*** = **24)**		**PD-ICBs (*****n*** = **28)**	
		***M***	***SD***	***M***	***SD***
*DPDT*	Delay discounting parameter *k* log_10_ transformed	−0.834	0.706	−1.053	0.675
	Probability discounting parameter *k* log_10_ transformed	−0.957	0.429	−0.981	0.466
*MCQ* ^∧^	*k* for all rewards	0.111	0.110	0.047	0.082
	*k* for small rewards	0.108	0.103	0.068	0.078
	*k* for medium rewards	0.107	0.108	0.042	0.042
	*k* for large rewards	0.098	0.104	0.043	0.043

MCQ, Monetary choice questionnaire; DPDT, Delay and probability discounting task.

∧ Seven participants (one PD + ICBs and six PD-ICBs) failed to complete MCQ.

Finally, delay and probability discounting were highly correlated, *r* = 0.597, *n* = 51, *p* < 0.01.

#### 3.2.3. Reactive aggression

Table 4 displays participants' performance on aggression (*PSAP* and *BPAQ*) measures.

5. PSAP. Three separate ANCOVAs were performed to compare PD + ICBs' and PD-ICBs' proportion of reward, retaliatory, and protective responses on *PSAP*. PD-ICBs opted for reward responses significantly more than PD + ICBs [*F*_(1,24)_ = 13.418, *p* = 0.001, η^2^ = 0.359]. PD + ICBs' proportion of retaliatory responses was significantly higher than PD-ICBs [*F*_(1,24)_ = 9.332, *p* = 0.005, η^2^ = 0.280]. No difference between the groups emerged for the proportion of “protect” choices on the *PSAP* [*F*_(1,24)_ = 0.404, *p* = 0.531].
6. BPAQ. Five separate ANCOVAs were run on overall *BPAQ* and its four dimensions. We found PD + ICBs to have significantly higher overall scores [*F*_(1,24)_ = 4.463, *p* = 0.045, η^2^ = 0.157] and higher physical aggression [*F*_(1,24)_ = 5.160; *p* = 0.032, η^2^ = 0.177] than PD-ICBs. There was a trend toward higher verbal aggression in PD + ICBs [*F*_(1,24)_ = 4.092; *p* = 0.054, η^2^ = 0.146]. No differences emerged for the anger [*F*_(1,24)_ = 0.957; *p* = 0.338] or hostility [*F*_(1,24)_ = 0.846; *p* = 0.367] subscales.

**Table 4:** Groups' performance on antisociality measures.

		**PD** + **ICBs (*****n*** = **16)**		**PD-ICBs (*****n*** = **21)**	
		***M***	***SD***	***M***	***SD***
*PSAP*	Proportion of A responses	24.554	13.398	54.700	19.016
	Proportion of B responses	40.997	19.568	18.630	16.319
	Proportion of C responses	34.240	17.466	26.670	12.107
*BPAQ* ^∧^	Total	65.310	12.488	59.500	12.534
	Physical aggression	15.81	4.102	14.500	4.883
	Verbal aggression	13.31	3.114	12.350	2.498
	Anger	14.88	5.353	13.750	3.768
	Hostility	21.31	7.031	18.900	5.32
*Stoplight*	Proportion of decisions not to brake	12.109	7.295	12.351	9.503
	Proportion of crashes	9.570	8.026	11.012	10.626
	Proportion of decisions to brake on yellow	73.828	15.304	67.113	21.117
	Proportion of decisions to brake on red	3.906	5.291	8.631	10.025
	Proportion of decisions to brake on red but crash occurred	0.586	1.260	0.893	1.752
*DBQ* ^∧^	*Total*	19.56	19.072	17.950	12.988
	*Lapses*	6.250	5.983	5.750	4.363
	*Errors*	3.812	4.215	3.950	2.856
	*Ordinary violations*	6.313	7.106	5.900	7.122
	*Aggressive violations*	3.188	4.520	2.300	3.131

∧ One PD-ICBs failed to complete the BPAQ and DBQ.

#### 3.2.4. Risk driving behavior

Table 4 displays participants' performance on risky driving (*Stoplight* and *DBQ*) measures.

7. *Stoplight*. No outcome measure showed any differences between PD + ICBs and PD-ICBs (all *p* > 0.094).
8. DBQ. ANCOVA revealed no group differences in overall *DBQ* scores [*F*_(1,24)_ = 0.025, *p* = 0.876] nor any subscales (all *p* > 0.454).

Finally, accounting for heightened reactive aggression in PD + ICBs, neither regression yielded any significant predictors of aggression, although hard task choices under medium probability (*b* = 1.809, *p* = 0.075, semi-partial correlation coefficient = 0.508) and delay discounting trended in the expected positive direction (*b* = 0.838, *p* = 0.080, semi-partial correlation coefficient = 0.498) for retaliatory responses on the *PSAP*.

## 4. Discussion

The present study allows us to objectivate that (a) patients suffering from PD + ICBs present higher levels of the “Positive Urgency” trait; (b) the incentive motivation and the choice impulsivity can be considered neuropsychological markers of ICBs induced by DRT in patients with PD; and, finally, (c) PD + ICBs patients manifest greater reactive aggression. Conversely, no differences emerged in the occurrence of risky driving behaviors, depressive symptoms, and anxiety, in these two groups of patients with PD.

First of all, we would focus our attention on the first piece of evidence. To the best of our knowledge, this is the first study in which impulsivity has been deeply evaluated in the two groups of patients with PD who differed exclusively for ICBs manifestation.

In PD + ICBs patients, we observe higher levels of positive urgency. This is a specific aspect of the multifaced trait of impulsivity and represents the tendency to act rashly in response to extreme positive emotions. Positive urgency has been shown strictly linked to maladaptive levels of risk-taking (often characterizing ICBs), such as pathological gambling, sexual risk-taking, drug use, and alcohol use (Cyders et al., 2007). We speculate about the possibility that positive urgency could be the pre-disposing trait feature for developing ICBs when an individual undergoes DRT.

By objectively evaluating the incentive motivation, we found that ICBs status itself did not predict hard task choice: in fact, those choices were contingent on the level of reward probability and magnitude. These findings are consistent with previous studies, demonstrating a main group effect (Evans et al., 2006, 2010). Evans and collaborators (Evans et al., 2006, 2010) employed a different paradigm (the *Card Arranging Reward Responsivity Objective Test*) where PD + ICBs and PD-ICBs arranged cards over a number of trials and could receive a higher reward for their increased speed on the final trial. As expected, in both studies, PD + ICBs were significantly faster to perform the task on the final rewarded trial than on the preceding trials. Significant differences in speed might be expected on the final trial, as it represents the unique opportunity to receive a reward for the task. This contrasts with the *EEfRT* where a reward is potentially available on every trial. This paradigm allows us to observe group effects related to the exacerbated incentive motivation in specific conditions, for example, when the reward magnitude is high and reward probability is low. This is, indeed, what has been found. All three levels of reward probability, as well as reward magnitude, interact with ICBs status to be significant predictors of hard task choice: it could be considered a fundamental feature of incentive sensitization in PD + ICBs (Berridge and Robinson, 2016). Therefore, PD + ICBs tend to choose the “easy-task” only under the conditions of low probability and less reward (Salamone and Correa, 2012), but in all other probability conditions, they are fixedly sensitized on the monetary cue of higher value and pursued it at all costs, without regard for the expected value (reward magnitude × reward probability). This behavior could be related to the fact that dopaminergic medication in predisposed patients with PD increases risky choices on gambling tasks and, in general, in rewarding conditions, regardless of the gamble or reward value (Rutledge et al., 2015; Timmer et al., 2018). Moreover, this result supports the hypothesis that dopamine is poorly sensitive to evaluate the effort cost, which results to be the least considered variable in this decision-making process (Walton and Bouret, 2019).

In relation to choice impulsivity, the findings highlighted more pronounced delay discounting in PD + ICBs on the objective measure of choice impulsivity (*DPDT*). In contrast, no group differences emerged neither on the complementary probability discounting measure nor on the subjective measure of delay discounting (*MCQ*). A hyperbolic function describes both delay and probability discounting and the constructs are highly correlated (Richards et al., 1999), making these findings very interesting. It remains to be understood why the *MCQ* did not elicit group differences. Null findings on choice impulsivity in PD + ICBs have emerged since recent systematic reviews of the literature (Dawson et al., 2018; Martini et al., 2018). This study employed the *MCQ*, as the only study featured in our review that did not detect group differences in choice impulsivity (Joutsa et al., 2015). Neither in these studies nor in our study, participants gained a reward on the *MCQ*, rendering it purely a measure of individual differences. Both Housden et al. (2010), with a moderately sized sample, and Voon et al. (2011) with a relatively large sample size, demonstrated that the MCQ could distinguish PD + ICBs and PD-ICBs without requiring an incentive. Alternatively, it might be unreasonable to expect objective and subjective measures of choice impulsivity to converge, as they might be measuring different constructs (Cyders and Coskunpinar, 2012). It is perhaps most prudent to simply conclude that this particular neurocognitive process requires further scrutiny, particularly when expressed in terms of probability discounting or hypothetical preferences. Contextual influences (e.g., the way probability discounting choices are framed to resemble risky choices to a greater or lesser degree) may be important in these latter cases (Lempert and Phelps, 2016).

We offer the first evidence of heightened reactive aggression in PD + ICBs. While it requires replication in a larger sample with a different aggression paradigm, such as the one proposed by Beyer et al. (2017), our data cohere with a previous study examining altruistic punishment in PD (Djamshidian et al., 2011). These authors found that PD + ICBs patients were more sensitive to norm violation and meted out more punishment at a personal cost when “on” compared to when “off” their medication, whereas there was no medication effect in PD patients without ICBs. The authors suggested that this result could reflect both altruistic and aggressive motivations. The action of “Striking back” in the PSAP task might reflect similar motivations in the present study: the PD + ICBs patients, driven to protect their points, could simultaneously punish their “antagonist,” to react to the loss of the rewards they accumulated.

We were unable to demonstrate any link between exacerbated incentive motivation and choice impulsivity and heightened reactive aggression in PD + ICBs, although there were positive trends in the expected direction. We might have merely lacked statistical power (Italian PD + ICBs *n* = 16). Trait incentive motivation predicts laboratory aggression and self-reported antisocial behavior (Seibert et al., 2010; Bacon et al., 2018); the importance of appetitive processes in aggressive behavior has been demonstrated in animal models (Golden et al., 2017); and heightened choice impulsivity relates to impulsive-antisocial traits (Hosking et al., 2017) and criminal behavior (Akerlund et al., 2016; Lee et al., 2017). This is not intended to suggest that these processes would operate in parallel in driving reactive aggression; they may interact, as it has been proposed in the case of ICBs (the “incentive salience of intertemporal choice model”) (Lades, 2012).

Finally, we did not find evidence of risky driving in PD + ICBs. A stronger investigation of this reported phenomenon would require the use of a driving simulator, with appropriate performance-based incentives, rather than relying on *Stoplight* or *DBQ*. Furthermore, we have to highlight how in patients with PD an increased risky driving behavior is often due to other crucial aspects, such as attentive dysfunctions, impairment of visuospatial functions, and motor slowness (Ranchet et al., 2020): controlling these co-factors could require a distinction among different groups of patients.

The general limitations of this investigation must be noted. First of all, we acknowledge that the sample size is low and underpowered, which may have influenced results, potentially leading to false positives. Despite this limitation, our study sample, design, and findings are highly novel. As such, these findings should be considered preliminary and in need of replication. Another limitation concerns the fact that the numerous rating scales used for this study overlap in the features that influence their scores and so will not be totally independent of one another. In further studies, a principal component analysis could help to determine how many of these ratings are truly orthogonal. Despite previous results suggesting the *GDS* was sufficiently sensitive to serve as a proxy measure of apathy (Sinha et al., 2013), we lacked a specific measure of this motivational aspect, which could be an important co-variate to study. We also lacked a measure of psychotic symptoms, which would have allowed us to control for any paranoia driving retaliatory responses on the *PSAP*. Similarly, we neither take a measure of negative (e.g., fatigued and stressed) states that could have impacted participants' performance on tasks, nor the time elapsed since the last medication administration.

The present study aligns with new directions in antisociality research focused less on negative affect (e.g., frustration, stress, and pain) (Berkowitz, 1998) and more on appetitive motivation (Golden et al., 2017) and aberrant cost–benefit decision-making (Hosking et al., 2017). This represents the first empirical exploration of impulsivity traits and antisocial behavior in PD + ICBs and complements our primary goal of clarifying the potential neurocognitive mechanisms underlying ICBs in PD. Deepening the knowledge about the neurocognitive impairments due to dopaminergic medications is essential for disease management, both in terms of drug dosage optimization and, when ICBs occur, to define targeted and effective cognitive-behavioral therapies. Finally, such insights would also serve to identify the most appropriate (i.e., sensitive) neurocognitive tasks to deploy in large-scale longitudinal studies aimed at generating predictive models of ICBs development in PD (Smith et al., 2016).

In summary, we have provided important evidence indicating that in patients with PD presenting positive urgency trait, the medications-induced dopaminergic overflow could alter the information processing, thus creating an imbalance between the incentive of reward and the costs due to effort, probability, and delay of the reward itself. We depict a condition that could be defined as “motivational myopia,” in which the reward salience overshadows the costs needed to reach a given goal. Extensions of our findings are still requiring larger cross-cultural samples and correlation with objective instrumental and clinical signs (e.g., PET scans, beta-cortical oscillations, tremor, and rigidity), as we need attempts to create a computational model able to unify these processes into a single incentive salience of intertemporal choice model (Lades, 2012). Looking forward, future studies should also better evaluate the burden of aggressive behaviors related to neurologic and psychiatric diseases and those affecting the general population, even comparing them in different countries.

## Data availability statement

The raw data supporting the conclusions of this article will be made available by the authors, without undue reservation.

## Ethics statement

Ethics approval was obtained from the Monash University Human Research Ethics Committee (CF16/129-2016000054) and the provincial Ethics Committee in Italy (Comitato Etico interaziendale delle Province di Lecco–Como–Sondrio). The patients/participants provided their written informed consent to participate in this study.

## Author contributions

AD, PO, AC, GF, and MY constructed the idea and the hypothesis for this research. AC, PO, DF, and MY planned methodology to reach the conclusion. AD, PO, DF, AC, GF, and MY organized and supervised the course of the project. AD, AC, PO, DF, and MY took responsibility for the construction of the whole or body of the manuscript. ND, AE, YC, and VL reviewed the article before submission for its intellectual content. All authors contributed to the article and approved the submitted version.
