# Supplementary material for: Motivational and myopic mechanisms underlying dopamine medication-induced impulsive-compulsive behaviors in Parkinson's disease
Source: Front Behav Neurosci. 2023 Jan 18;16:949406. doi: 10.3389/fnbeh.2022.949406 (PMC9889554; doi:10.3389/fnbeh.2022.949406)
Supplement: Supplementary file 1 [file Table_1.DOCX]

| **AREA OF INTEREST** | **COMPUTERIZED TASKS** | **AUTOADMINISTERED COMPUTERIZED QUESTIONNAIRES** |
| --- | --- | --- |
| INCENTIVE MOTIVATION | *Effort Expenditure for Rewards Task* - *EEfRT*  (for a detailed task description, see Treadway et al., 2009; for psychometric properties, see Horan et al., 2015; Reddy et al., 2015). | *Behavioural Inhibition Scale and Behavioural Approach Scale - BIS/BAS*  (see Carver & White, 1994) |
| CHOICE IMPULSIVITY | *Delay and Probability Discounting Task - DPDT*  (for a detailed task description see Richards et al., 1999; for psychometric properties, see Hamilton et al., 2015). | *Monetary Choice Questionnaire - MCQ*  (see Kirby et al., 1999; for psychometric properties, see Hamilton et al., 2015). |
| REACTIVE AGGRESSION | *Point Subtraction Aggression Paradigm - PSAP*  (for detailed task description and psychometric properties, see Cherek et al., 1997). | *Buss-Perry Aggression Questionnaire -BPAQ*  (see Buss & Perry, 1992; for psychometric properties, see Gerevich et al., 2007). |
| RISKY DRIVING | *Stoplight Task*  (for detailed task description and psychometric properties, see Chein et al., 2011) | *Driver Behavior Questionnaire - DBQ*  (see de Winter & Dodou, 2010; Lajunen, Parker, & Summala, 2004 |

*Incentive motivation.*

*Effort Expenditure for Rewards Task* (*EEfRT*) (for a detailed task description, see **Treadway et al., 2009**; for psychometric properties, see **Horan et al., 2015; Reddy et al., 2015**).

*EEfRT* requires participants on each trial to decide between an ‘easy-task’ (and potentially receive $1 for completion) and a ‘hard-task’ (and potentially receive between $1.20-$4.30 for completion) for 20 minutes. The ‘easy-task’ requires pressing the L button on a keyboard (if right-handed) with the index finger 30 times within 7 seconds. The ‘hard-task’ requires pressing the S button with the fourth finger 100 times within 30 seconds. Probability of reward delivery varies randomly across trials: there are low (12%), medium (50%) and high (88%) reward probability trials. Any reward earned is revealed after each trial, two of which are randomly selected for payment.

*BIS/BAS* (see **Carver & White, 1994**)

*BIS/BAS* is a 24-item subjective measure of two motivational systems: behavioural inhibition (*BIS*) and behavioural approach (*BAS*). *BAS* is comprised of drive, fun seeking and reward responsiveness subscales. The drive subscale (e.g., ‘*I go out of my way to get things I want*’) was used as a subjective measure of incentive motivation.

*Choice impulsivity.*

*Delay and Probability Discounting Task (DPDT)* (see **Richards et al., 1999**; for psychometric properties, see **Hamilton et al., 2015**).

*DPDT d*ynamically modifies (smaller/sooner or smaller/certain) and (larger/later or larger/uncertain) choices until the number of delay and probability indifference points are determined for each participant. Delays range from 2 to 365 days, while probabilities ranged from 90 to 25%. The larger later/uncertain reward amount was always $10 while smaller sooner/certain reward amounts varied. Participants face choices resembling ‘*Would you prefer $3.50 now or $10 in 2 days?*’ One choice is randomly selected for participant payment.

*Monetary Choice Questionnaire (MCQ)* (see **Kirby et al., 1999**; for psychometric properties, see **Hamilton et al., 2015**).

*MCQ* is a 27-item questionnaire poses choices between hypothetical smaller-soon sums and larger-later sums (e.g., *Would you prefer $14 today or $25 in 19 days?*). Larger-later sums can be small ($25-$35), medium ($50-60) or large ($75-85). Participants were not reimbursed for completing this questionnaire.

*Reactive aggression.*

*Point Subtraction Aggression Paradigm (PSAP)* (for detailed task description and psychometric properties, see **Cherek et al., 1997**).

In *PSAP*, participants are instructed that they are playing against an anonymous online opponent (who is actually fictitious). At each trial, participants face three choices: press ‘A’ 100 times to earn one point (10 cents); press ‘B’ 10 times to deduct a point from their ‘opponent’ (but not receive the point themselves); or press ‘C’ 10 times to protect their accumulated points for a short period of time. The aim is to accumulate as many points as possible. As participants complete their presses for each trial, the computer randomly and obviously deducts points from their total under the guise of the ‘opponent’ ‘stealing’ points. Participants’ proportion of ‘B’ responses is of key interest. Participants were paid for their accumulated points. No participant expressed doubts about the reality of the ‘opponent’.

*Buss-Perry Aggression Questionnaire (BPAQ)* (see **Buss & Perry, 1992**; for psychometric properties, see **Gerevich et al., 2007**).

*BPAQ* is a 29-item subjective measure of four dimensions of aggression: physical aggression (e.g., ‘*Given enough provocation, I may hit another person’*); verbal aggression (e.g., ‘*When people annoy me, I may tell them what I think of them’*); anger (e.g., ‘*When frustrated, I let my anger show’*); and hostility (e.g., ‘*I am suspicious of overly friendly strangers’*).

*Risky driving.*

*Stoplight Task* (see **Chein et al., 2011**).

*Stoplight* is a simple driving task in which participants must pass through 32 intersections to reach their target destination in under eight minutes. Participants observe a car moving along a road and, as intersections with yellow traffic lights approach, can brake and stop temporarily using the space-bar or proceed through the intersection, but risk crashing and losing a significant amount of time. When exactly lights change to yellow is unpredictable, as is likelihood of oncoming traffic. There was no reward provided for reaching the target destination on time.

*Driver Behavior Questionnaire (DBQ)* (for detailed description and psychometric properties, see **de Winter & Dodou, 2010**; **Lajunen, Parker, & Summala, 2004**).

*DBQ* is a 27-item measure of general driving behaviour. It has four subscales: lapses (e.g., *How often do you hit something when reversing that you had not previously seen?*), errors (e.g., *How often do you fail to check your rear-view mirror before pulling out?*), ordinary violations (e.g., *How often do you overtake a slow driver on the inside?*) and aggressive violations (e.g., *How often do you sound your horn to indicate your annoyance to another road user?*).

*Covariates.*

We measured (legal and illicit) substance abuse, depression, anxiety and trait impulsivity with the following well-established scales: *Alcohol Use Disorder Identification Test* (**Bohn et al., 1995**), *Drug Abuse Screening Test-10* (**Skinner, 1982**)*, *Fagerström Test for Nicotine Dependence* (**Heatherton et al., 1991**), *Geriatric Depression Scale-15* (**Shiekh et al., 1986**), *Parkinson Anxiety Scale* (**Leentjens et al., 2014**)* and Urgency, Premeditation (lack of), Sensation Seeking, Positive Urgency, Impulsive Behavior Scale *UPPS-P* (**Cyders et al., 2007**).
